# Primary cutaneous B‐Cell lymphoblastic lymphoma presenting with solitary scalp mass in a female child: A case report and review of the literature

**DOI:** 10.1002/ccr3.6553

**Published:** 2022-11-06

**Authors:** Fatemeh Montazer, Alireza Sanei Motlagh, Ramtin Dastgir

**Affiliations:** ^1^ Pathology Iran University of Medical Sciences Tehran Iran; ^2^ Mazandaran University of Medical Sciences Sari Iran; ^3^ Faculty of Dentistry, Tehran Medical Sciences Islamic Azad University Tehran Iran

**Keywords:** B‐cell origin, CD20, CD45, CD79a, CD99, cutaneous lymphoblastic lymphoma, Ki67, scalp mass, TdT

## Abstract

Lymphoblastic lymphoma is a group of non‐Hodgkin lymphomas that account for approximately 2% of all lymphomas. This is a report of a case of a young girl presenting with a solitary scalp mass which was resected. Histopathological examination of the mass along with bone marrow analysis revealed primary cutaneous B‐cell lymphoblastic lymphoma. A nine‐year‐old girl presenting with an asymptomatic erythematous, non‐tender scalp mass present for 12 months was admitted. Skull and brain were intact and devoid of any pathological findings on computed tomography imaging. Systemic examination also showed no evidence of mass lesion in other parts of the body. The lesion was resected and referred for pathological analysis. Microscopic study revealed heavy diffuse dermal and subcutaneous infiltration of monomorphous medium‐sized mononuclear cells, with fine chromatin, scant cytoplasm, and variable nucleoli along with intact epidermis and presence of grenz zone. Tumor cells dissect through the collagen fibers. Extensive mitotic figures and focal infiltration of the skin adnexa are seen. IHC study revealed that TdT, CD79a, CD99, CD45, CD20, and Ki67 markers were positive. According to these findings, a definitive diagnosis of primary cutaneous lymphoblastic lymphoma of B cell type was concluded. The 1‐year follow up after necessary treatment revealed normal findings without traces of recurrence. Lymphoblastic lymphomas (LBL) are a neoplasm of immature B cells belonging to the B‐(B‐LBL) or T‐cell lineage (T‐LBL) that accounts for approximately 2% of all lymphomas. Lymphoblastic lymphoma (LBL) is similar to acute lymphoblastic leukemia (ALL) and the differentiation between these neoplasms is based upon proportion of involvement of lymphoblasts in bone marrow. It has a higher male to female predominance, higher incidence in older children and younger adults, and a relatively higher frequency of CNS and gonadal involvement. The differential diagnosis is based on immunohistochemistry study of B‐cell linage tumor markers. Cutaneous involvement is present in about one third of patients with B‐LBL but rarely in patients with ALL.

## Graphical abstract

Despite the rarity of the disease, primary cutaneous lymphomas are heterogeneous group of non‐Hodgkin lymphomas that account for 2% of all lymphomas. In most cases, skin lesions are secondary to or concomitant with bone, or lymph node involvement. Furthermore, cutaneous involvement at the time of presentation is usually rare.

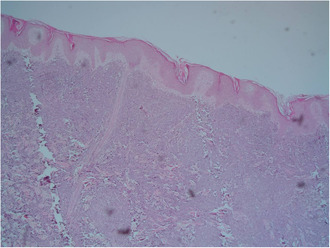

## 1 INTRODUCTION

Primary cutaneous lymphomas are heterogeneous group of non‐Hodgkin lymphomas with the origin of T‐ or B‐cells and account for approximately 2% of all lymphomas. In tissue sections of B type lymphoblastic lymphoma (B‐LBL), generally a diffuse pattern of growth is noted.^1^, ^2^ B‐LBL is always positive for B‐cell markers CD19, CD79a, and CD22. CD10, CD24, PAX5, and terminal deoxytransferase (TdT) are expressed in most cases, while the expression of CD20 and CD34 is variable and CD45 may be absent.^3^ The common criteria to differentiate lymphoblastic lymphoma (LBL) from acute lymphoblastic leukemia (ALL) are their manifestations as bulky masses in solid organs, and focal or absent bone marrow involvement. LBL presents with skin involvement more frequently. Furthermore, the incidence of central nervous system involvement in LBL is higher than ALL.^4^ B‐LBL is more frequently associated with skin or subcutaneous tissue involvement or bone lesions, while T‐LBL predominantly shows mediastinal involvement. It usually occurs in early middle age, involves lymph nodes and extra‐nodal sites, and usually does not invade the bone marrow.^5^, ^8^ Here, we report a case of cutaneous lymphoblastic lymphoma with the origin of B‐cells presenting with solitary scalp mass in a female child.

## 2 CASE PRESENTATION

**FIGURE 1:**
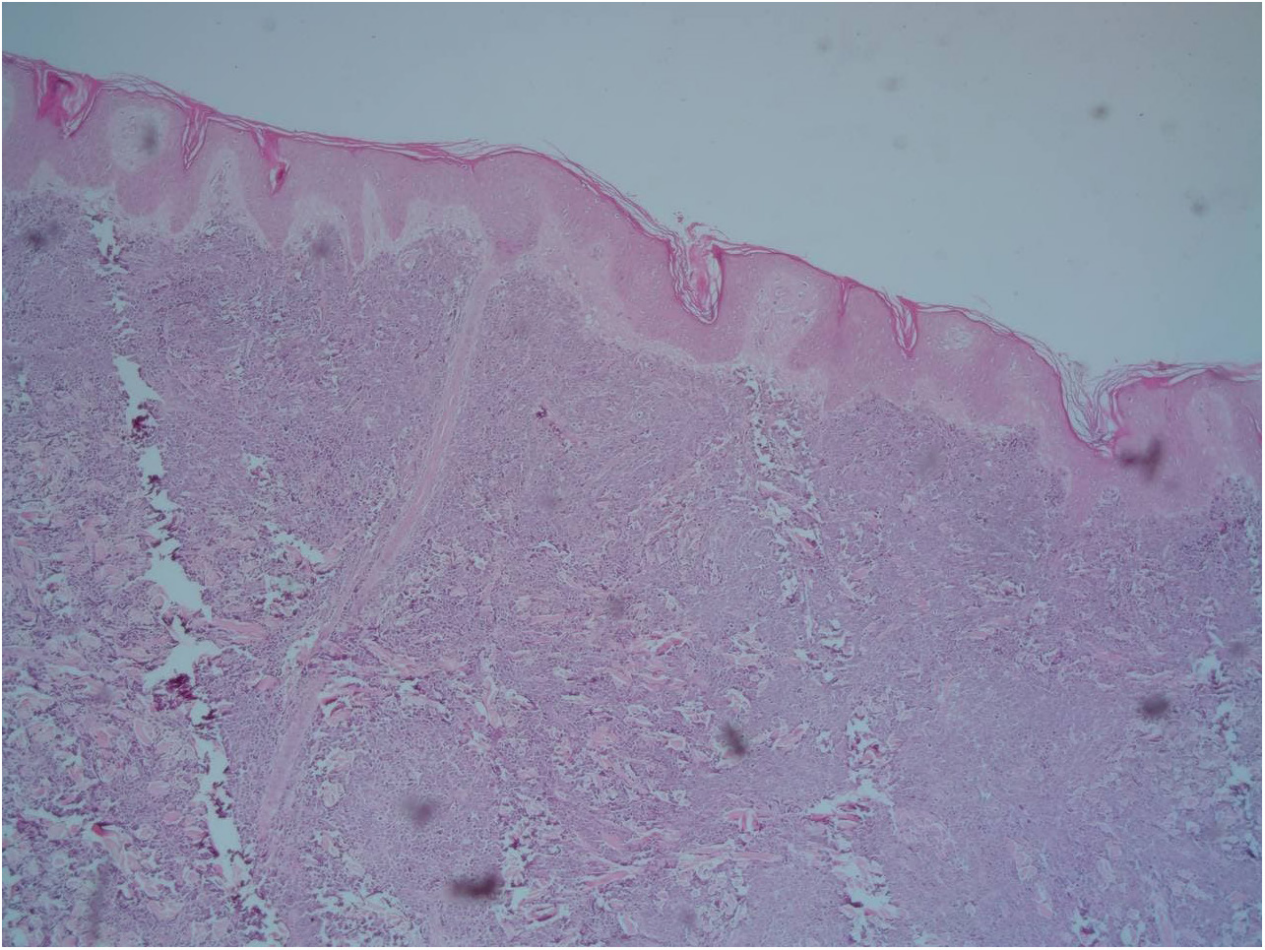
Hematoxylin and eosin (H&E) staining, depicting dense dermal infiltration of monomorphous medium‐sized mononuclear cells. The epidermis remained intact. (200×).

**FIGURE 2:**
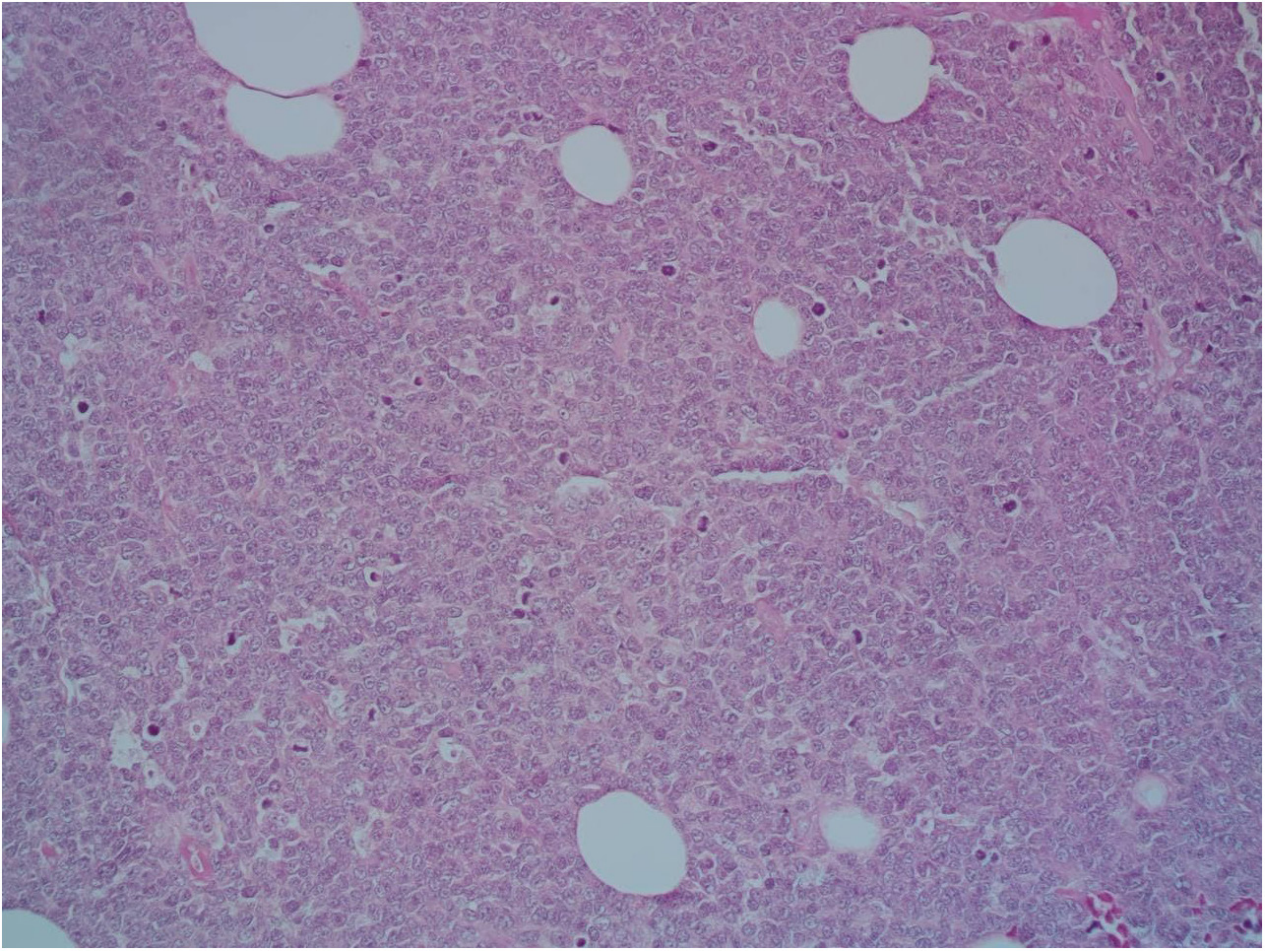
Hematoxylin and eosin staining, infiltration of monomorphous medium‐sized mononuclear cells, with fine chromatin, scant cytoplasm, and small nucleoli. Mitotic figures were frequent (400×).

**FIGURE 3:**
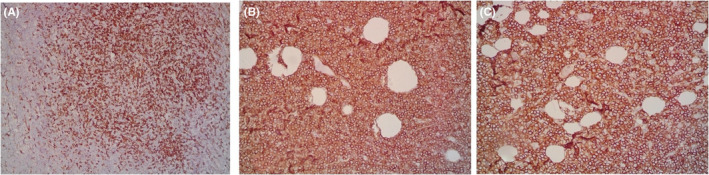
Immunohistochemistry study:CD45 (A), CD79a (B), CD99 (C), markers are positive in tumor cells (x200).

**FIGURE 4:**
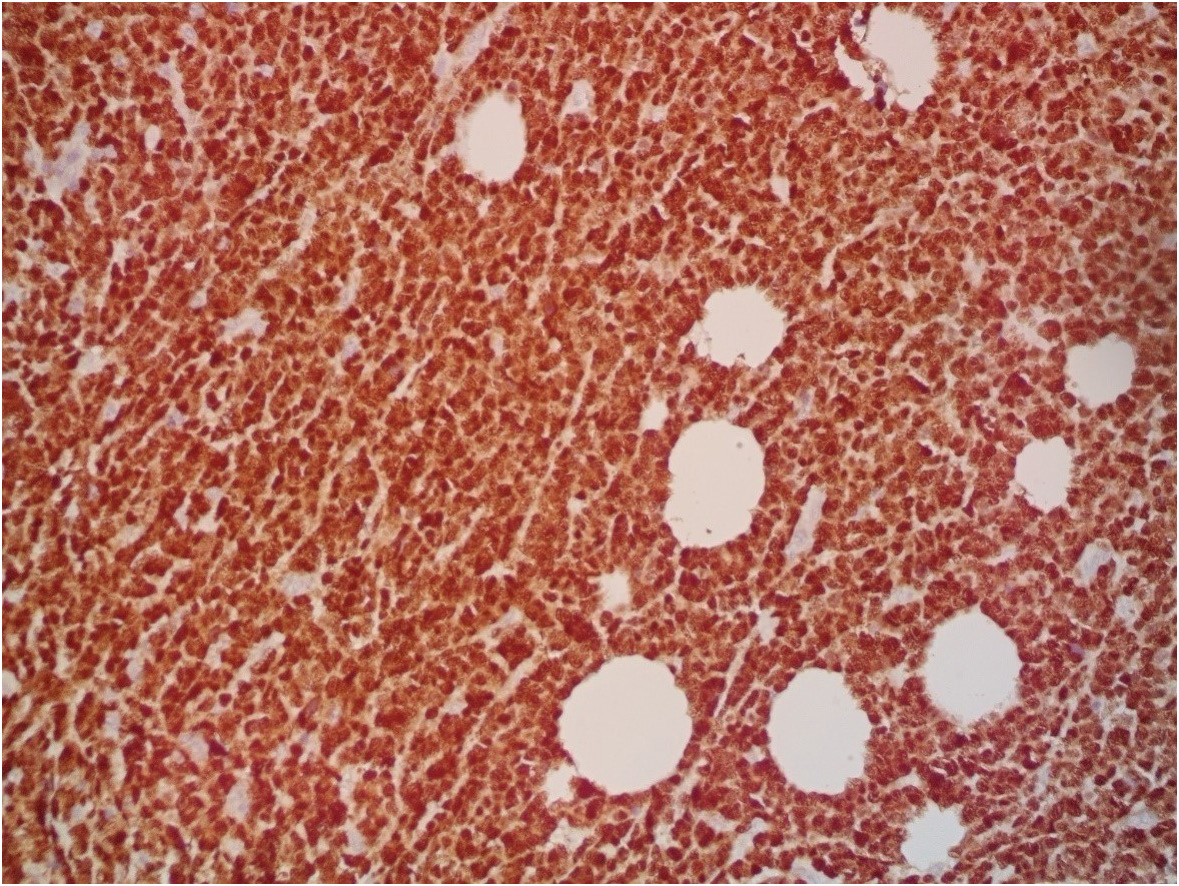
Immunohistochemistry study: TdT marker is strongly positive in tumor cells. (400×).

**FIGURE 5:**
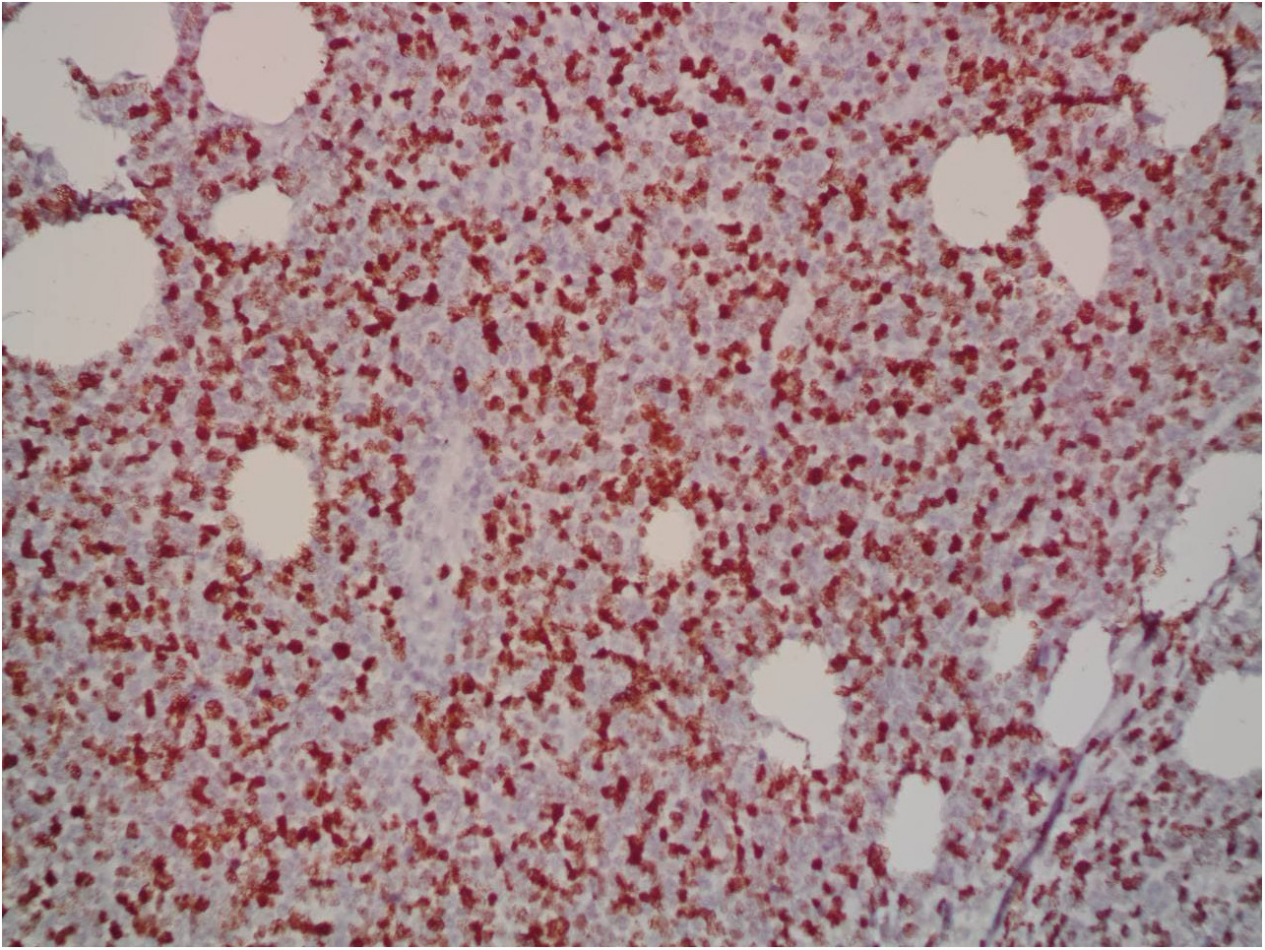
Immunohistochemistry study: Ki67 expression in approximately 40% of tumor cells (400×).

A 9‐year‐old Iranian, Caucasian girl with the chief complaint of a solitary mass present on her scalp for almost 12 months with no past medical history, no history of blood transfusion, and no recurrent infection was admitted to hospital. A painless, erythematous, dome‐shaped mass with well‐defined borders and firm consistency with the size of 4.5 × 3 × 1 cm was noted on physical examination. No pruritus or tenderness of lesion was evident. The patient did not complain of fever or weight loss during recent months. Complete blood count (CBC) findings were normal, with no sign of cytopenia or leukocytosis. Lactate dehydrogenase (LDH) value was in normal range. Peripheral blood smear (PBS) demonstrated no significant findings and only shows mild anisocytosis of red blood cell. Furthermore, no blast or atypical lymphocyte is observed. Cerebrospinal fluid analysis yielded normal findings. Bone marrow biopsy (BMB) and bone marrow aspiration (BMA) showed normocellular marrow with orderly maturation. Moreover, evidence of lymphocytosis or immature cell (blast) was not observed. Computed tomography scan (CT‐scan) of the brain revealed no involvement of skull or brain tissue. Systemic examination further revealed no evidence of mass lesion in other parts of the body. All internal organs including liver, spleen and intra‐abdominal lymph nodes had normal findings. Laboratory evaluations revealed normal findings. Hence, *en bloc* resection was achieved and the lesion referred to laboratory for further pathological study. Microscopic analysis revealed heavy diffuse dermal and subcutaneous infiltration of monomorphous medium‐sized mononuclear cells, with fine chromatin, scant cytoplasm, and small nucleoli. Mitotic figures were frequent and tumor cells were observed infiltrating between collagen bundles. The integrity of epidermis remained intact (Figures 1 and 2). Based on immunohistochemistry (IHC)analysis, CD99, TdT, and CD79a markers were strongly positive in tumor cells with diffuse pattern. Furthermore, CD45 and CD20 markers were also positive in some of the tumor cells (Figures 3 and 4). Ki67 marker was positive in approximately 40% of tumor cells (Figure 5). Tumor cells were negative for MNF116, EMA, CK7, CK20, Melan A, HMB45, TTF1, S100, Chromogranin, NSE, CD3, CD56, and desmin. Bone marrow biopsy revealed that the tumor had not involved the marrow and full‐body CT‐scan and whole‐body bone scan was devoid of any pathological findings. Therefore, the definitive diagnosis of primary cutaneous lymphoblastic lymphoma of B cell type was concluded. One‐year follow‐up of the patient after proper treatment revealed normal findings and no trace of recurrence.

## 3 DISCUSSION

Lymphoblastic lymphomas (LBL) are a neoplasm of immature B cells belonging to the B‐(B‐LBL) or T‐cell lineage (T‐LBL) that account for approximately 2% of all lymphomas and for approximately 8% of all lymphoid malignancies.^5^ LBL is more prevalent in males compared to females, with a male/female prevalence of 1.4. The highest rate of occurrence was reported in children younger than 15 years old with a rate of 3.6 per 100,000; followed by 0.8 per 100,000 in patients aged between 25 and 64 years old; and then increased to 1.7 per 100,000 in the oldest age group of cases older than 65 years old.^2^ It commonly develops in children and young adults and presents as multiple purple nodules or papules within the head and neck region. Most cases have no constitutional symptoms.^6^ In most cases, skin lesions are secondary to or concomitant with bone, bone marrow, or lymph node involvement. Furthermore, cutaneous involvement at the time of presentation is usually rare which further fortifies the rarity of our case.^7^ Differential diagnosis of LBL include blastoid variant of mantle cell lymphoma, Burkitt lymphoma or myeloid leukemia which may arise in some particular adult cases.^2^ Histological presentations are usually sufficient in order to distinguish lymphoblastic from mature B‐ or T‐cell neoplasms; however, the key for diagnosis is the characterization of immunophenotype by flow cytometry.^8^ Lymphoblastic lymphoma (LBL) is similar to acute lymphoblastic leukemia (ALL) and the differentiation between these neoplasms is based upon proportion of involvement of lymphoblasts in bone marrow (<25% for lymphoblastic lymphoma and >25% for ALL).^9^, ^10^ It has a higher male to female predominance (except female preponderance in B‐type) with a ratio of 1.4:1,^5^ higher incidence in older children and younger adults, and a relatively higher frequency of CNS and gonadal involvement in the course of the disease. With the same chemotherapy regimen, patients with B‐LBL have more complete remission (CR) and rarely develop leukemia compared with those with ALL. Furthermore, the survival rate is higher in patients who had a complete response to chemotherapy than that of patients with partial or no response to chemotherapy.^11^, ^12^ In terms of histopathological characteristics, cutaneous lymphoblastic lymphoma shows heavy infiltrates of atypical cells among collagen bundles with scant cytoplasm and prominent nucleoli. A focal or diffuse starry‐sky pattern may be present. Cutaneous involvement is present in 33% of patients with B‐LBL and 1% of patients with B‐cell ALL that is considered as leukemia cutis.^13^ Immunohistochemistry is necessary to rule out possible differential diagnosis including leukemic infiltration of acute myeloid leukemia (AML), T‐cell ALL, Ewing sarcoma, primitive neuroectodermal tumor, neuroblastoma, small cell melanoma, Merkel cell carcinoma, and Burkitt lymphoma. Utilizing IHC staining for Bcl‐2, Bcl‐6, and TdT, LBL can be differentiated from Burkitt lymphoma. Bcl‐6 is negative in LBL, while Bcl‐2 and TdT are negative in Burkitt lymphoma.^14^, ^15^

## 4 CONCLUSION

Despite the rarity of this disease and the presence of different treatment modalities for LBL, a few general conclusions can be made including: the modern therapy of LBL should follow the same principles of ALL therapy; taking into account the scarce results of salvage therapy and the optimal results obtained with frontline therapy of pediatric patients a comparable therapeutic approach should be adopted in all patients regardless of age; a regimen including 5 g/m^2^ MTX blocks could be part of a highly effective modality, in which MRT could be safely omitted and low‐dose cranial irradiation should be delivered only to patients with advanced forms of the disease. Furthermore, seeking for stronger prognostic indicators, the presence of adverse (onco)‐genetic abnormalities and the early evaluation of CT/PET and MDD/MRD in the future could allow a more rational, risk‐oriented use of MRT, SCT, and new‐targeted therapies.^2^

## AUTHOR CONTRIBUTIONS

F.M was responsible for data collection and revision of the manuscript. A.SM was responsible for preparation of early draft of the manuscript. R.D was responsible for final composition and revision of the manuscript. All authors contributed equally to the manuscript.

## FUNDING INFORMATION

No funding was received for this article.

## CONFLICT OF INTEREST

The authors declare no conflict of interest.

## ETHICAL APPROVAL

This study was performed according to the principles outlined by the World Medical Association's Declaration of Helsinki on experimentation involving human subjects. This study has been further approved by the ethics committee of Mazandaran University of Medical Sciences.

## CONSENT

Written informed consent was obtained from the patient's parents to publish this report in accordance with the journal's patient consent policy.

## Data Availability

Data sharing is not applicable to this article as no new data were created or analyzed in this study.
